# Cross-talk between cardiac lymphatics and immune cells regulates inflammatory response and cardiac recovery after myocardial infarction

**DOI:** 10.3389/fimmu.2025.1557250

**Published:** 2025-05-20

**Authors:** Zhihua Yang, Zeyu Zhang, Shaoling Feng, Xujin Ning, Liuli Guo, Yijia Du, Shuai Wang, Xianliang Wang, Jingyuan Mao

**Affiliations:** ^1^ Department of Cardiovascular Diseases, First Teaching Hospital of Tianjin University of Traditional Chinese Medicine, Tianjin, China; ^2^ National Clinical Research Center for Chinese Medicine Acupuncture and Moxibustion, Tianjin, China; ^3^ Institute of Traditional Chinese Medicine, Tianjin University of Traditional Chinese Medicine, Tianjin, China

**Keywords:** cardiac lymphatics, immune cell, myocardial infarction, cardiac recovery, lymphangiogenesis, myocardial fibrosis

## Abstract

Myocardial infarction (MI) is a life-threatening disease with high morbidity and mortality, closely associated with immune-inflammatory responses. As essential pathways for immune cell clearance and interstitial fluid drainage, lymphatic vessels are critical in regulating tissue fluid homeostasis and systemic immune surveillance. Cardiac lymphatics interact with immune cells, directly and indirectly, to mediate post-MI inflammation, participate in the clearance of necrotic tissue, and contribute to cardiac remodeling. Studies indicate that after MI, promoting cardiac lymphangiogenesis can accelerate the clearance of infiltrated immune cells, reduce the production of pro-inflammatory cytokines, improve myocardial edema, mitigate inflammatory responses and fibrosis, and support recovery of cardiac function. Meanwhile, immune cells regulate the structure and function of cardiac lymphatics, influencing lymphangiogenesis and drainage efficiency. The interaction between cardiac lymphatics and immune cells is crucial for myocardial repair post-MI. This review first systematically summarizes the structure and function of cardiac lymphatics, then sorting the relationship between cardiac lymphatics and immune cells and their roles in myocardial repair after MI and finally proposes therapeutic strategies targeting the interaction between cardiac lymphatics and immune cells in MI treatment, to provide prospective insights for the prevention and treatment of MI in the future.

## 1 Introduction

Acute myocardial infarction (AMI), or myocardial infarction (MI), is the leading cause of death among cardiovascular diseases and is characterized by necrosis of heart muscle tissue due to prolonged ischemia and hypoxia of the coronary arteries (1). AMI is one of the highest mortality diseases worldwide, with an increasingly younger patient population posing a severe threat to human health (2). According to the World Health Organization (WHO), more than 23.3 million people will die from cardiovascular disease by 2030 (3). In recent years, pharmacological thrombolysis, coronary intervention, and coronary artery bypass grafting have been effective in revascularizing occluded coronary arteries and reducing early mortality from AMI (4). However, some patients continue to experience adverse ventricular remodeling due to extensive myocardial necrosis or impaired repair of infarcted areas, leading to heart failure (HF) and, in severe cases, cardiac rupture. Improving these patients’ prognoses remains a significant clinical challenge (5). Studies have shown that MI is closely linked to the immune-inflammatory response, with excessive infiltration of immune cells at sites of myocardial injury exacerbating inflammation, increasing infarct size, and, in severe cases, leading to HF (6, 7). Therefore, inhibiting immune cell-mediated inflammatory responses has become an essential strategy for mitigating AMI-induced injury (8, 9).

The lymphatic system, an integral part of the circulatory system, has garnered increased research attention in recent years with the discovery of lymphatic markers and the advancement of lymphatic functional imaging and quantification techniques. These developments have highlighted the critical role of cardiac lymphatics in the pathophysiology of heart disease (10, 11). Unlike the closed, high-pressure, and circular nature of the vascular system, the lymphatic system operates as an open, low-pressure, unidirectional network that transfers extracellular fluid to the venous system, playing an essential role in tissue fluid homeostasis, lipid absorption from the digestive tract, and systemic immune surveillance (12). Cardiac lymphatics are essential for cardiac health. Promotion of cardiac lymphangiogenesis can improve cardiac function following MI (13), but insufficient lymphatic expansion contributes to the development of HF after MI (13). Studies have shown that post-MI lymphangiogenesis facilitates immune cell clearance, accelerates the resolution of inflammation, aids in repairing infarct-induced cardiac injury, and mitigates pathological ventricular remodeling (14). Thus, enhancing therapeutic lymphangiogenesis has emerged as a promising new strategy for treating MI. During MI, interactions between cardiac lymphatics and immune cells significantly influence each other.

On the one hand, lymphatics serve as pathways for the clearance of immune cells and inflammatory mediators post-MI, expediting the removal of inflammatory cells. Conversely, different immune cell populations play distinct roles in promoting or inhibiting lymphangiogenesis and lymphatic remodeling. The cross-talk between cardiac lymphatics and immune cells is essential for myocardial repair after MI, and understanding this relationship is critical for deciphering the role of cardiac lymphatics in post-MI inflammatory response and myocardial repair. Summarizing the fundamental pathological changes in cardiac and lymphatic systems post-MI can provide disease-specific therapeutic strategies. Moreover, understanding the regulatory mechanisms of cardiac lymphangiogenesis and the effects of immune cells on lymphangiogenesis and myocardial repair could identify improved therapeutic targets for the condition. This paper provides a comprehensive overview of these topics to deepen our understanding of how the lymphatic system influences disease progression after MI. Additionally, it offers new insights into the interactions between immune cells and cardiac lymphatics in myocardial repair following injury, paving the way for effective clinical strategies to stimulate cardiac lymphangiogenesis for treating heart disease.

## 2 Cardiac lymphatics

### 2.1 Structure of cardiac lymphatics

**Figure 1:**
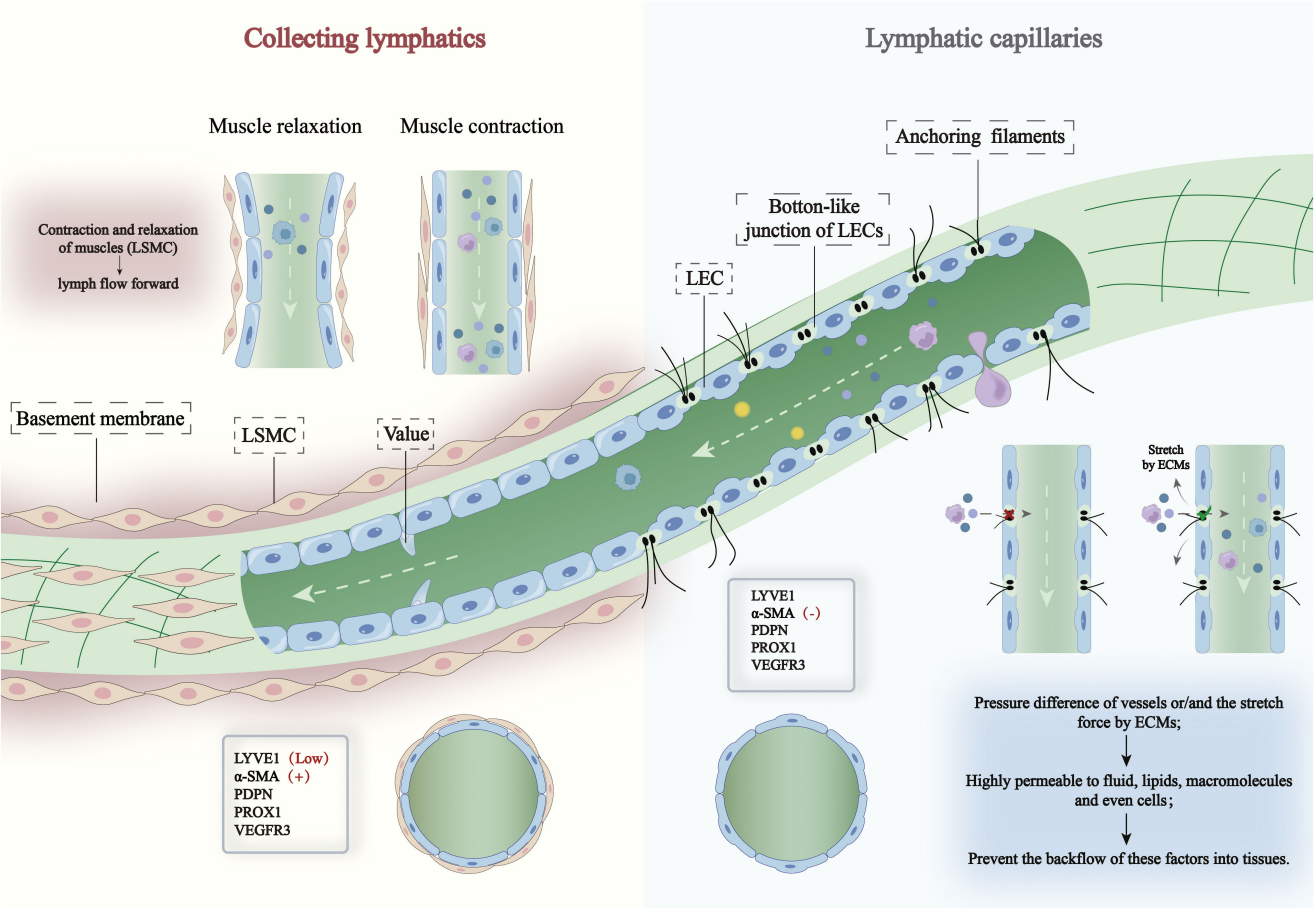
Structure and function of cardiac lymphatic vessels. The structure of cardiac lymphatic vessels is composed of capillary lymphatics and collecting lymphatics. Capillary lymphatics are lined with a single layer of continuous lymphatic endothelial cells (LECs), which are connected by “button-like” junctions and have high permeability. Collecting lymphatics are formed by the convergence of capillary lymphatics, with thin walls and larger diameters, and the cells are connected in a “zipper-like” fashion, preventing the permeation of fluids and cells. Within the collecting lymphatics, there are semilunar valves that prevent lymphatic backflow and are a primary marker of the transition from capillary lymphatics to collecting lymphatics. When there is a pressure difference across the vasculature or/and the stretching force of the extracellular matrix (ECM) on LECs, the permeability of capillary lymphatics increases, allowing fluids, lipids, and large molecules to enter the capillary lymphatics, while also preventing these factors from reflowing.

The lymphatic system, a vital immune defense network within the human body, is widespread and involved in immune responses and tissue homeostasis. Lymphatic vessels are an essential component of this system, consisting mainly of capillary lymphatics and collecting lymphatics (15), each with distinct morphology, cellular structures, and functions (**Figure 1**) (16). Capillary lymphatics, the starting points of the lymphatic network, reside within tissue interstitial spaces and begin as enlarged blind-ended vessels. Their walls are formed by overlapping oak-leaf-shaped monolayers of lymphatic endothelial cells (LECs), which express surface markers specific to lymphatics, such as Podoplanin (PDPN), Prospero homeobox 1 (PROX1), and Lymphatic vessel endothelial receptor-1 (LYVE-1) (17). The LECs are loosely arranged with significant intercellular gaps, forming “overlapping” or “button-like” connections, and are often absent of a continuous basement membrane. Capillary lymphatics lack smooth muscle cells or pericyte coverage but feature thin anchoring filaments attached to the vessel walls and surrounding extracellular matrix. These filaments maintain vessel openness during increased interstitial pressure (18, 19) and prevent capillary collapse (20).

In contrast to capillary lymphatics, collecting lymphatics exhibit “zipper-like” tight junctions between LECs, and their walls are covered by lymphatic smooth muscle cells (LSMCs) that express α-smooth muscle actin (αSMA), which imparts contractile capability, along with a continuous basement membrane (21) valves within the lumens of collecting lymphatics create a “pump-like” structure, ensuring lymph flows in a unidirectional manner (22). Unlike the cardiovascular system, the lymphatic system lacks a central pump. Instead, lymph flow relies on alternating contraction and relaxation of LSMCs, propelling lymph fluid through valves to reach upstream lymph nodes and ultimately return to the venous system (23, 24). Additionally, these vessels interact with capillaries to facilitate antigen presentation and immune cell transport.

### 2.2 Function of cardiac lymphatics

The lymphatic system, an integral component of the circulatory system, connects with blood vessels, forming a refined vascular network throughout the human body and closely linking with microcirculation (25). Lymphatic vessels play critical roles in reverse cholesterol transport, immune cell clearance, and interstitial fluid drainage, critical for maintaining tissue fluid homeostasis, lipid absorption in the digestive tract, and systemic immune surveillance (12). In the heart, the lymphatic system uses capillary lymphatics to expel excess interstitial fluid, absorb extravasated cells and macromolecules, and transport them unidirectionally via collecting lymphatics to draining lymph nodes, the thoracic duct, and the inferior vena cava, ultimately returning tissue fluid to the bloodstream—one of its essential physiological functions (15). The function of lymphatic vessels is maintained primarily through the activity of LECs. Lymphangiogenesis, or lymphatic vessel formation, is a critical process in maintaining the typical structure and function of the lymphatic network in physiological and pathological states. This process involves LECs’ migration, proliferation, and tube formation (26). Under normal physiological conditions, LECs are relatively quiescent. However, chemokines in pathological states such as chronic inflammation and lymphedema prompt LECs to migrate to local tissues, where they proliferate under the influence of growth factors to form new lymphatic vessels. Functional lymphatic vessels provide an efficient pathway to drain excess interstitial fluid, immune cells, inflammatory cells, and mediators caused by inflammation, thereby reducing interstitial edema and vascular inflammation (27).

Conversely, inadequate lymphatic drainage can result in interstitial lymphedema, where impaired lymphatic return and forward propulsion lead to excessive fluid accumulation, triggering immune cell infiltration and inflammatory cascades and exacerbating endothelial dysfunction induced by immune inflammation (28). Therefore, the lymphatic network is essential for maintaining tissue fluid balance and immune surveillance (29). The heart, an organ prone to autoimmunity, contains a complex lymphatic network that collects macromolecules, proteins, electrolytes, and fluids from the interstitial space. It returns them to circulation, regulating tissue pressure and preventing edema (30). Numerous studies of both domestic and international indicate a strong correlation between cardiac lymphatic development or dysfunction and the progression of MI (14). Insufficient lymphangiogenesis or impaired lymphatic drainage compromises myocardial repair following MI (14).

## 3 Changes in cardiac lymphatics after MI

**Figure 2:**
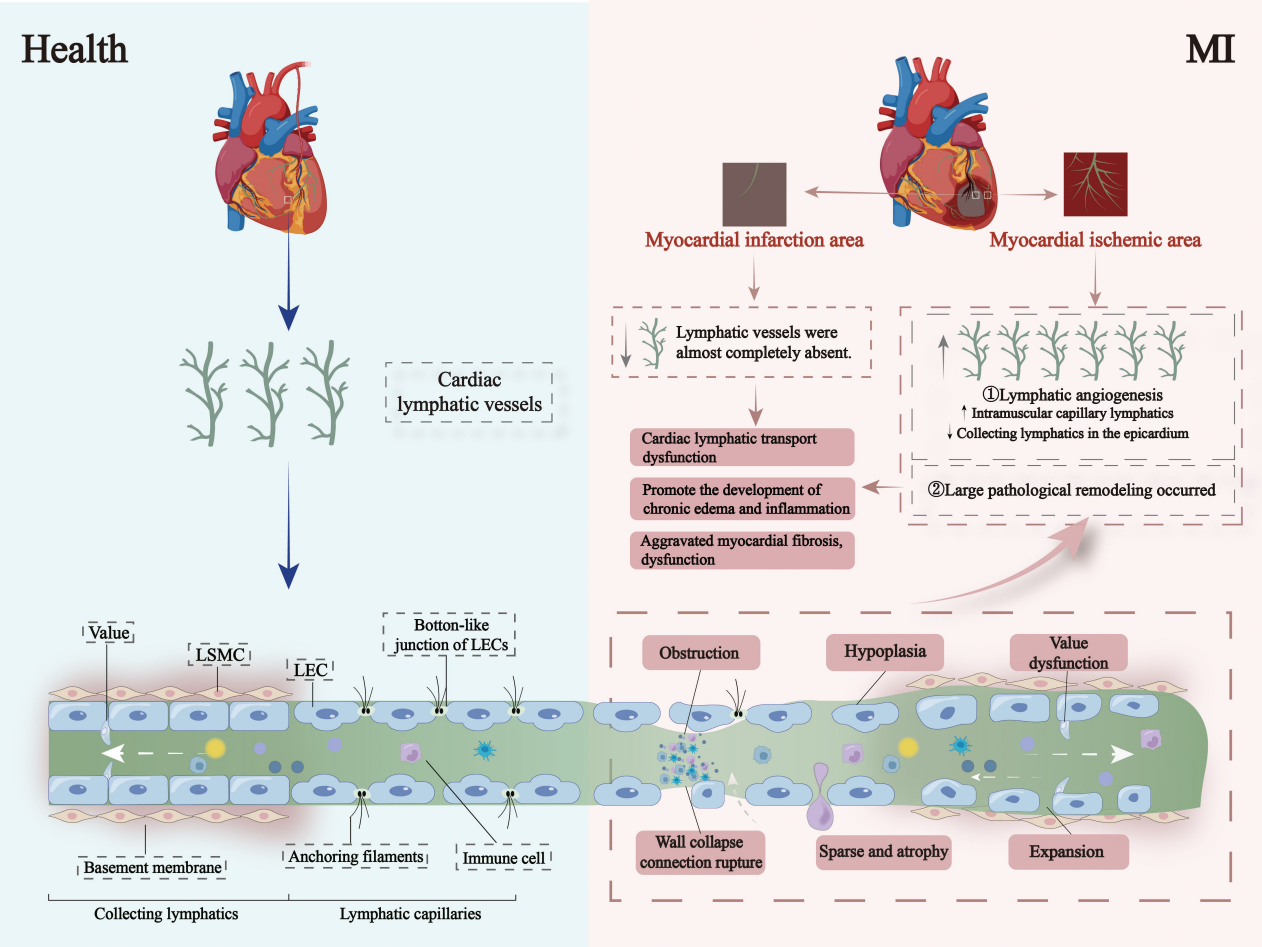
Changes of cardiac lymphatic vessels after MI. After MI, the lymphatic vessels in the infarcted area are almost completely absent, while the lymphatic vessels in the non-infarcted areas undergo extensive remodeling. Although MI can significantly induce the formation of initial lymphatic vessels in the heart, there is poor remodeling of epicardial pre-collecting and collecting lymphatic vessels, including sparse and atrophic lymphatic vessels in the infarcted area, underdeveloped lymphatic vessels in the non-infarcted area, dysfunction of lymphatic valves, abnormal lymphatic vessel proliferation, lymphatic vessel dilation, obstruction of capillary lymphatics, and collapse of capillary lymphatic walls with broken connections.

In adults, lymphatic vessels are typically quiescent under physiological conditions but can be reactivated under pathological conditions (10). Post- MI lymphangiogenesis is linked to increased immune cell infiltration, driven by pro-apoptotic signals released by damaged cells, cellular debris, and cytokines from adjacent cells (31). After MI, capillary lymphangiogenesis increases within the myocardium while the epicardial collecting and conduit lymphatics decrease. This reduces lymphatic transport capacity, negatively affecting fluid and inflammation clearance in infarcted and non-infarcted regions, thus exacerbating cardiac dysfunction (14). Clinically, myocardial edema has been observed to extend beyond the infarcted area following MI, indicating lymphatic dysfunction. In a study by Orianne Henri et al. (14), a rat MI model was established via ligation of the left anterior descending coronary artery. Their observations revealed nearly complete loss of lymphatic vessels in the infarcted area and pathological remodeling of lymphatic vessels in the non-infarcted regions. While MI significantly induced initial cardiac lymphangiogenesis, the epicardial pre-collecting and collecting lymphatics remained sparse and poorly remodeled, leading to impaired cardiac lymphatic transport. This dysfunction contributed to chronic myocardial edema, inflammation, increased fibrosis, and worsened cardiac function. Injection of recombinant human VEGF-C protein into infarcted myocardial areas has been shown to stimulate significant capillary lymphangiogenesis, reduce pathologically remodeled collecting lymphatic vessels, enhance overall lymphatic transport capacity, relieve myocardial edema, decrease fibrosis, and improve cardiac ejection function (10, 14). However, as noted earlier, the functional integrity of newly formed lymphatic vessels may not match that of pre-MI cardiac lymphatics. Moreover, studies in mice and zebrafish reveal early lymphatic dilation and increased lymphatic density shortly after MI (32). Changes of cardiac lymphatic vessels after myocardial infarction is shown in **Figure 2**.

## 4 The Role of immune cells in inflammatory response and myocardial repair post-MI

**Figure 3:**
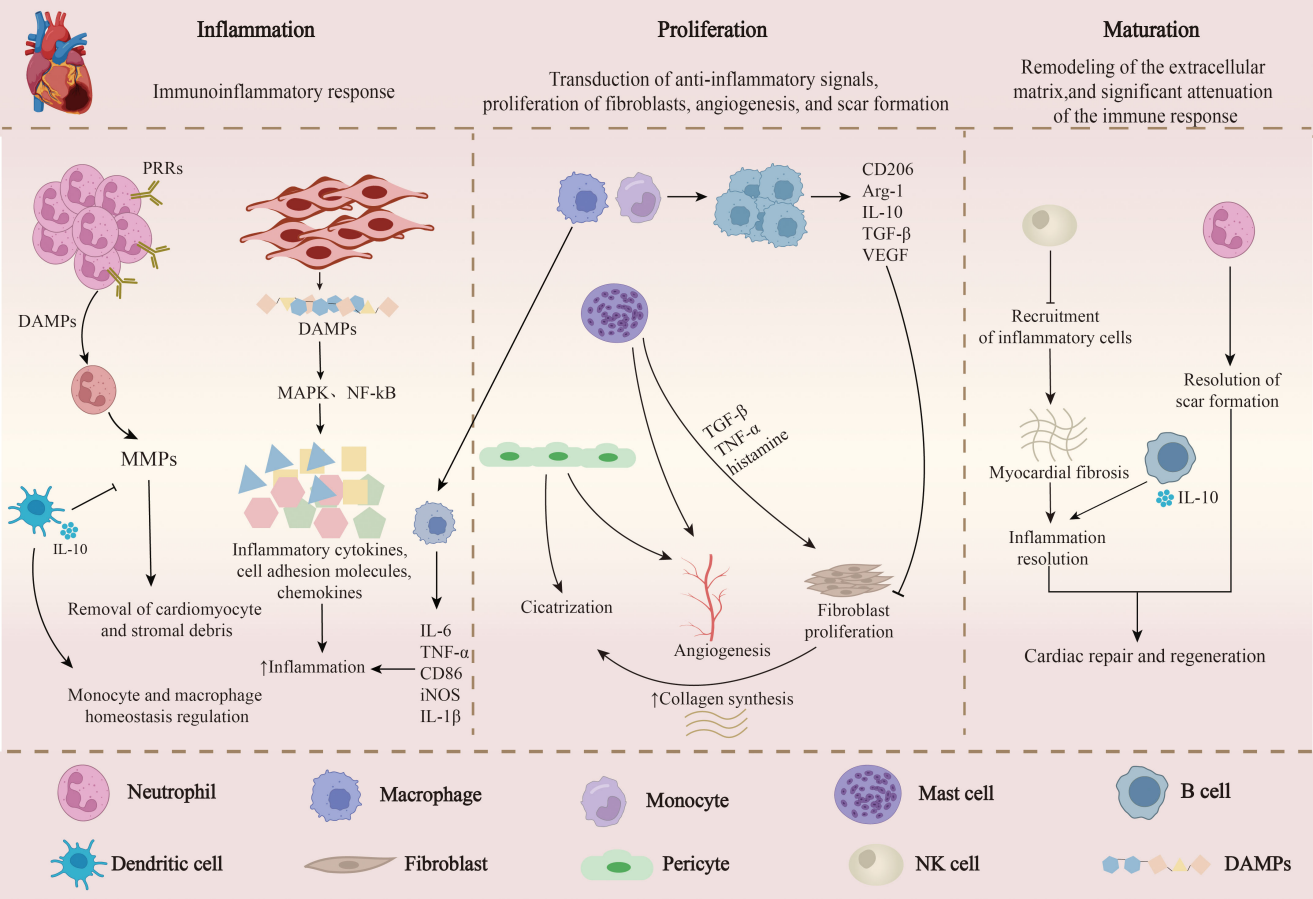
The role of immune cells in inflammatory response and myocardial repair post-MI. Post-myocardial infarction inflammation and myocardial repair involve multiple immune cells and are characterized by three main phases: inflammation phase, proliferation phase, and maturation phase. 1) Inflammation Phase: This phase is marked by a rapid influx of cytokines, chemokines, and immune cells to clear damaged tissue. Myocardial cells release Damage-Associated Molecular Patterns (DAMPs), activating the MAPK and NF-kB pathways, which induce inflammatory cytokines, chemokines, and cell adhesion molecules to amplify inflammation and initiate an inflammatory cascade reaction. DAMPs bind to Pattern Recognition Receptors (PRRs) on the surface of neutrophils, releasing Matrix Metalloproteinases (MMPs) to clear damaged myocardial cells and matrix debris. 2) Proliferation Phase: This phase is characterized by the transduction of anti-inflammatory signals, proliferation of fibroblasts, angiogenesis, and scar formation. M2 macrophages release anti-inflammatory factors to suppress the inflammatory response, reduce collagen deposition, and promote angiogenesis. Mast cells promote angiogenesis and scar formation by releasing cytokines and also regulate atrial myocardial remodeling. Some pericytes promote angiogenesis.3)Maturation Phase: This phase is characterized by the remodeling of the extracellular matrix, a significant decrease in immune response, cross-linking of collagen fibers, and apoptosis of immune cells, leading to the formation of stable scar tissue, which promotes cardiac regeneration and repair.

Inflammation is closely associated with the onset and progression of MI. After MI, necrotic and apoptotic cardiomyocytes activate damage-associated molecular patterns (DAMPs), releasing various cytokines and chemokines that recruit and activate diverse immune cells, contributing to myocardial repair post-MI (33). Various immune cells, cytokines, and chemokines regulate the inflammatory response and repair following MI. After MI, the body initiates a series of primary and adaptive immune-inflammatory responses and subsequent repair processes, the balance of which is critical for cardiac function recovery and patient prognosis. Ischemia-induced cardiomyocyte death in the infarcted region recruits and activates immune cells (neutrophils, macrophages, mast cells, and monocytes), leading to an inflammatory infiltration response (34), followed by collagen deposition, extracellular matrix remodeling, and scar formation (35). The role of immune cells in the initial pro-inflammatory and subsequent anti-inflammatory phases post-MI is vital and closely linked to the final infarct size and ventricular remodeling after MI; a prolonged and intense pro-inflammatory response may lead to adverse left ventricular remodeling and increased risk of HF (36). The inflammatory response and myocardial repair after MI involve multiple immune cells and proceed through three main phases: the inflammatory phase, the proliferative phase, and the maturation phase, each overlapping and progressively advancing (37). The inflammatory phase is marked by the rapid influx of cytokines, chemokines, and immune cells that clear damaged tissue; the proliferative phase is characterized by myofibroblast proliferation and collagen deposition to replace dead tissue, while collagen fiber cross-linking and immune cell apoptosis signify the final stabilization phase (38). During AMI, immune cell infiltration acts as a double-edged sword. The cardiac immune response to ischemic injury, marked by immune cell infiltration, involves not only neutrophils, monocytes/macrophages, and dendritic cells (DCs) but also B and T lymphocytes. These cells collaboratively coordinate the clearance of dead cardiomyocytes, participate in stabilizing and maturing scar tissue through granulation tissue formation, and secrete pro-angiogenic, anti-apoptotic, and anti-inflammatory mediators to regulate subsequent cardiac repair and inflammation resolution (**Figure 3**) (39).

### 4.1 Neutrophils

Neutrophils, the most abundant and rapidly responding innate immune cells in the body, play dual roles in pro-inflammatory and anti-inflammatory processes during the inflammatory phase following AMI. In the early phase of AMI inflammation, neutrophils infiltrate the infarct area, initiating local inflammation. Necrotic cardiomyocytes release DAMPs, which act as danger signals that bind to pattern recognition receptors on the surface of neutrophils. This activates the MAPK pathway and nuclear transcription factor NF-κB, inducing the production of inflammatory cytokines, chemokines, and cell adhesion molecules (primarily guided by NF-κB), which amplifies inflammation and initiates the inflammatory cascade response (40, 41). Guided by chemokines, integrins (CD11a/CD11b) and selectins (CD62L) on neutrophil surfaces bind tightly with intercellular adhesion molecules (ICAM)-1/ICAM-2 on endothelial cells. This facilitates neutrophil migration, recruiting large numbers of neutrophils to the infarct area. These neutrophils release matrix metalloproteinases and neutrophil elastase (NE), engulfing and clearing dead cardiomyocytes and matrix debris, thus promoting injury repair in the infarct zone (42). However, this clearance also leads to further damage. When neutrophil activity is prolonged, and phagocytosis is enhanced, activated neutrophils release complement proteins, matrix metalloproteinase-9 (MMP-9), myeloperoxidase (MPO), and NE through degranulation, producing large amounts of reactive oxygen species (ROS) and forming neutrophil extracellular traps (NETs), which induce cardiomyocyte necrosis and extracellular matrix (ECM) degradation, resulting in irreversible myocardial injury (42, 43). NETs, web-like structures released by neutrophils, mainly comprise DNA and histones with various granule proteins attached, including NE, MPO, cathepsin G, and leukocyte proteinase 3 (44). During acute myocardial infarction, excessive release of NETs amplifies the inflammatory response, leading to tissue damage and adverse cardiovascular events such as ventricular remodeling and arrhythmias (45). Studies show that plasma levels of dsDNA (a NET marker) are significantly elevated in AMI patients and positively correlate with infarct size (46). Pharmacological inhibition of Peptidyl arginine deiminase-4 significantly reduces neutrophil recruitment to the infarct area, suppresses inflammatory cytokine secretion, and NETs release, mitigating cardiomyocyte apoptosis, thereby decreasing infarct size and improving left ventricular ejection fraction (LVEF), as well as cardiac structure and function (47).

Similarly, DNase I degradation of NETs increases myocardial cell survival post-MI and improves left ventricular remodeling (48). *In vitro* studies further confirm that NETs promote apoptosis in neonatal rat cardiomyocytes, supporting NETs’ role in cardiomyocyte damage (49). Elevated NET levels following acute MI can mediate cardiomyocyte damage and fibroblast activation, exacerbating myocardial necrosis and fibrosis (50). Moreover, research reports that during the acute inflammatory phase post-MI, knocking down or pharmacologically inhibiting the expression of the triggering receptor expressed on myeloid cells-1 on neutrophils can reduce early, short-term neutrophil infiltration, thereby decreasing myocardial fibrosis and improving ventricular function (51).

### 4.2 Monocytes/macrophages

Macrophages are critical immune cells in MI patients, playing essential roles in cardiac function recovery across the stages of MI. In the early phase after AMI, monocytes derived from the bone marrow and spleen are rapidly recruited to the injured myocardium, where they differentiate into macrophages under the influence of chemokines. The monocyte/macrophage population is instrumental in the inflammatory and reparative processes following MI. Macrophages accumulate in large numbers in the infarct zone, where they not only help clear necrotic cells and debris but also secrete various cytokines and growth factors that affect cardiomyocyte survival, proliferation, and differentiation (52). In mice, monocytes are divided into Ly6C^high^ and Ly6C^low^, while macrophages are categorized into two major subsets. Pro-inflammatory M1 macrophages are involved in host defense, upregulating inducible nitric oxide synthase (INOS) and inflammatory factors such as Interleukins (IL), tumor necrosis factor (TNF)-α, chemokines, and MMPs. They contribute to inflammatory injury by enhancing phagocytosis and producing ROS. Reparative M2 macrophages, on the other hand, upregulate arginase-1 and anti-inflammatory factors, including VEGF and transforming growth factor-beta (TGF-β), promoting inflammation resolution and tissue repair (53, 54). Studies indicate that in the early stages post-MI, Ly6C^high^ monocytes, and M1 macrophages predominate in the infarcted myocardium. As inflammation subsides in the later stages, there is a phenotypic shift to Ly6C^low^ monocytes and M2 macrophages. These different phenotypes play distinct and critical roles following MI (55, 56).

### 4.3 Lymphocytes

Clinical studies have shown that a reduced lymphocyte count is independently associated with a higher incidence of mortality in AMI patients. Activated regulatory T cells (Tregs) can inhibit cardiac remodeling post-MI by inducing macrophage differentiation into the M2 phenotype by upregulating arginase-1, IL-13, osteopontin, and TGF-β expression (57). Conversely, Treg depletion can accelerate dilation and exacerbate ventricular remodeling following MI (58). There is also evidence suggesting that myocardial injury can trigger the accumulation of B lymphocytes. Yan et al. (59) first reported on the dynamic changes in myocardial immune cells in mice following ischemic injury. Their findings showed a marked increase in B lymphocytes one day after MI, peaking at day 7, with elevated levels persisting even at day 14 post-MI. Studies indicate that, following AMI, mature B lymphocytes infiltrate the infarcted myocardium and contribute to the inflammatory response by secreting chemokine CC motif ligand 2 (CCL2) and CCL7. These chemokines, in turn, induce Ly6C^high^ monocytes to migrate from the bone marrow into the infarcted myocardium, amplifying the inflammatory response, exacerbating myocardial injury, and impairing cardiac function (60).

### 4.4 Dendritic cells

As essential antigen-presenting cells, DCs regulate various inflammatory cells involved in innate and adaptive immunity, playing a crucial role in immune responses (61). DCs offer protective effects during the inflammatory and subsequent healing processes post-MI. In MI models with DCs depletion in mice, a marked increase in Ly6C^high^ monocytes and M1 macrophages infiltrated both the infarcted and surrounding areas and a notable decrease in Ly6C^low^ monocytes and M2 macrophages. The expression of inflammatory cytokines and MMP-9 is elevated, while the expression of anti-inflammatory cytokine IL-10 drops significantly. These findings suggest that DC depletion intensifies the inflammatory response and extracellular matrix degradation by activating inflammatory monocytes and M1 macrophages while inhibiting anti-inflammatory monocytes and M2 macrophages, thus delaying post-MI cardiac healing and worsening cardiac function (62). Autopsy studies in ST-elevation MI patients have shown that, in cases of cardiac rupture compared to non-rupture cases, there is increased infiltration of CD68^+^ macrophages, with reduced CD209^+^ and CD11c^+^ DCs infiltration and reparative fibrosis, suggesting that a decrease in DCs alongside increased macrophage infiltration impairs reparative fibrosis, raising the risk of cardiac rupture post-MI. This indicates the protective role of DCs in post-MI inflammation and subsequent healing processes (63).

Immune cells play a crucial role in the early inflammatory response and the later myocardial repair process following MI. Targeting immune cell recruitment in the early phase of MI and appropriately modulating the inflammatory response may help selectively inhibit damage and promote repair, thereby improving clinical outcomes (64, 65). Immunomodulation has emerged as a promising therapeutic strategy.

## 5 Cardiac lymphangiogenesis promotes myocardial repair after MI

The heart contains an extensive network of capillary lymphatics, which drain lymph fluid via the subepicardial pre-collecting lymphatic vessels to the aortic and paratracheal mediastinal lymph nodes. This extensive lymphatic network is essential for maintaining normal cardiac function and facilitating myocardial healing after injury. In recent years, accumulating evidence has highlighted the crucial role of cardiac lymphangiogenesis in repairing MI-induced cardiac damage. Poor myocardial tissue repair following MI is a primary cause of chronic HF and mortality, presenting significant clinical treatment challenges. Cardiac lymphangiogenesis is a critical process in heart repair, which can reduce myocardial damage, improve cardiac function, and prevent HF post-MI (66). When lymphangiogenesis is insufficient or maladaptive, lymphatic transport dysfunction occurs, leading to chronic interstitial edema, inflammatory response, and eventually interstitial fibrosis (31). Studies have shown that inflammatory cells exudated after myocardial infarction produce vascular endothelial growth factors VEGF-C and VEGF-D, increasing VEGFR expression in nearby lymphatics. This promotes lymphangiogenesis in the infarct border and infarct zones, draining interstitial fluid and metabolic byproducts of hypoxic tissue, thereby reducing cardiac edema and improving heart function (32). During granulation tissue formation post-MI, lymphatic vessels play a role by draining excess proteins and fluids, aiding in fibrosis maturation and scar tissue formation, and providing a suitable microenvironment for potential myocardial regeneration (67). Animal studies indicate insufficient lymphatic drainage worsens myocardial edema and inflammation, thereby exacerbating myocardial injury (68). Furthermore, administering VEGF-C significantly promotes lymphangiogenesis in the myocardium after MI in mice, markedly improving left ventricular ejection fraction and cardiac function (69). LYVE-1 is a specific lymphatic marker, and LYVE-1 knockout mice show aggravated fibrosis and pathological ventricular remodeling after MI, with progressively deteriorating cardiac function, underscoring the critical role of lymphangiogenesis in post-MI cardiac function. Myocardial fibrosis is the primary pathological process leading to HF (70). Cardiac lymphangiogenesis regulates pathological processes such as inflammation and edema, promoting the clearance of inflammatory factors, relieving inflammation, reducing edema, and inhibiting myocardial fibrosis (71). When lymphangiogenesis is inadequate or maladaptive, lymphatic transport dysfunction ensues, resulting in chronic interstitial edema and inflammatory response, ultimately leading to interstitial fibrosis (31).

## 6 Cross-talk between cardiac lymphatics and immune cell regulates cardiac recovery after MI

### 6.1 Cardiac lymphangiogenesis facilitates immune cell clearance post-MI

The lymphatic system, a part of the circulatory system, plays an integral role in fluid homeostasis and immune surveillance by providing pathways for immune cell clearance and tissue fluid drainage. The cardiac lymphatics maintain fluid balance, control inflammation, and support myocardial repair through fibrotic or regenerative responses (12). Dysfunction in lymphatic structure and function post-AMI and impaired lymphatic drainage lead to the buildup of inflammatory cytokines, immune cells, and tissue fluid, contributing to myocardial edema and excessive inflammation, thereby exacerbating myocardial damage and fibrosis (11). Increased cardiac lymphatic density correlates with improved cardiac function and enhanced lymphangiogenesis post-MI can increase immune cell transport (69). When immune cell transport and lymphatic clearance are impeded, viable myocardium suffers, scar formation intensifies, and cardiac output declines significantly (31). Studies have shown that following MI, circulating monocytes and activated macrophages heavily infiltrate the heart, accompanied by cardiac lymphangiogenesis. Newly formed lymphatic plexuses display co-localization with immune cells (69). Macrophages and dendritic cells adhere and transport via LYVE-1 receptors on lymphatic endothelial cells, and MI models lacking LYVE-1 display significantly higher myeloid cell counts (31, 72). VEGF-C intervention notably increases cardiac lymphangiogenesis while reducing leukocyte infiltration, particularly myeloid cells, demonstrating that post-MI cardiac lymphangiogenesis promotes inflammatory cell clearance (14). Although lymphatic remodeling occurs across the acute and chronic phases of ischemic heart disease, the altered configuration of subepicardial collecting lymphatics may hinder therapeutic needs. Clinical studies indicate that myocardial VEGF-C injection promotes capillary lymphangiogenesis, reduces myeloid cell infiltration, maintains the transport function of collecting lymphatics, reduces cardiac edema and fibrosis, and ultimately improves cardiac function (14). Cardiac lymphatics thus offer channels for immune cell drainage, alleviating myocardial edema and supporting ventricular remodeling, ultimately enhancing cardiac function. Targeting cardiac lymphangiogenesis could be a preventive and therapeutic strategy for cardiovascular diseases. Stimulating lymphangiogenesis to facilitate inflammatory cell clearance is promising for AMI treatment and may provide a novel approach to repair injured myocardium post-MI.

### 6.2 Impact of immune cells on cardiac lymphatic structure and function post-MI

**Figure 4:**
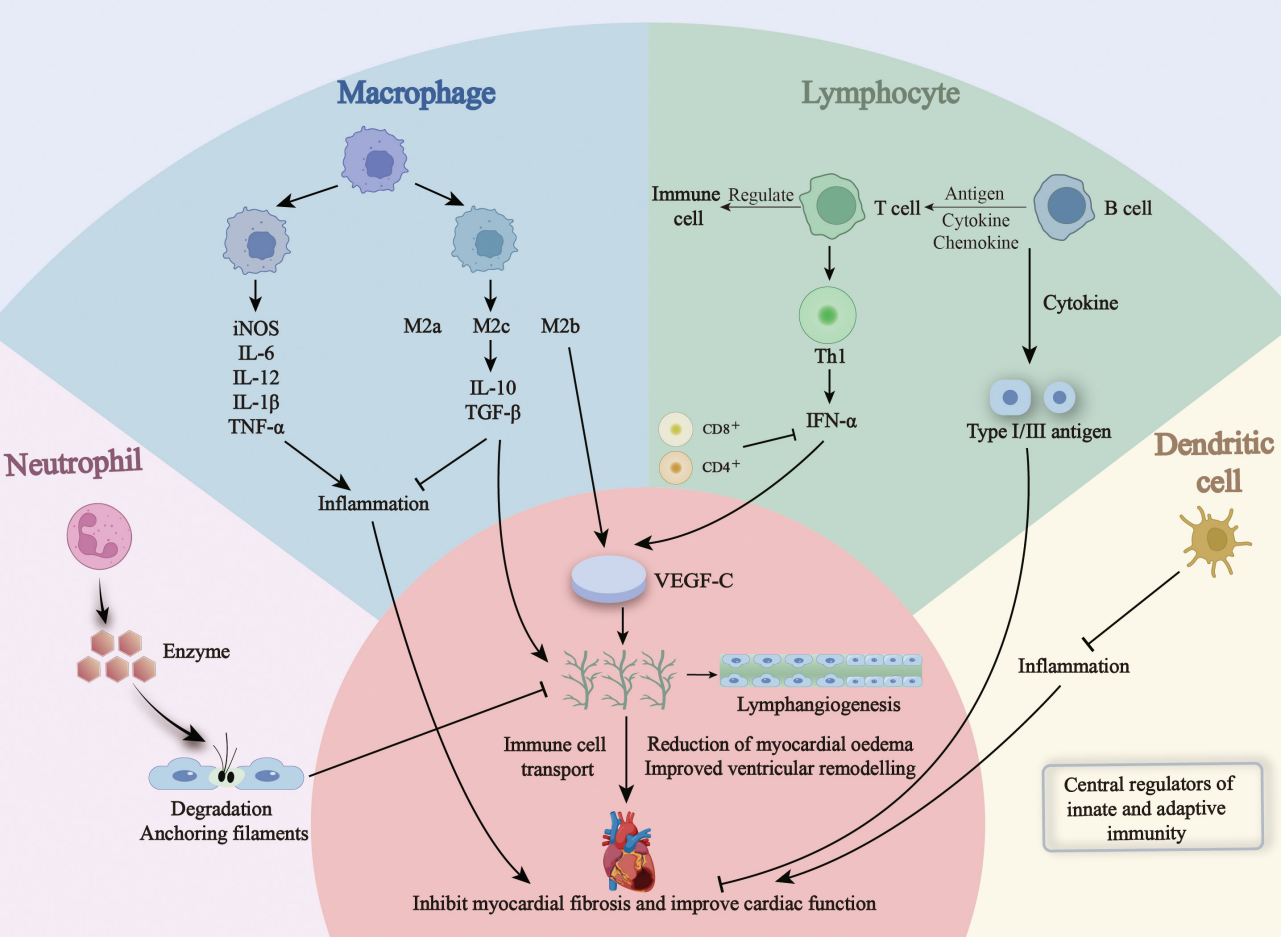
Cardiac lymphatics interact with immune cells in myocardial repair after Ml. Cardiac lymphatic vessels provide a conduit for the drainage of immune cells and inflammatory factors, and in turn, immune cells exert either a promoting or inhibitory effect on the growth and survival of cardiac lymphatic endothelial cells, influencing the structure and function of cardiac lymphatics. 1) During inflammation, neutrophils release enzymes (such as neutrophil elastase MPO) that degrade Emilin-1 anchoring filaments, leading to impaired lymphatic transport and exacerbated myocardial edema; 2) Macrophages can differentiate into two subtypes: pro-inflammatory (M1) and anti-inflammatory (M2) phenotypes. M1 macrophages secrete pro-inflammatory factors such as inducible nitric oxide synthase (iNOS), tumor necrosis factor (TNF)-α, IL-1β, and IL-6, which exacerbate inflammation and myocardial damage. M2 macrophages primarily secrete anti-inflammatory factors like TGF-β and IL-10, playing roles in anti-inflammation, neovascularization, and tissue repair. 3) B lymphocytes present antigens to T cells and produce cytokines and chemokines, ultimately regulating the function of other immune cells and potentially promoting the expression of myocardial type I and III collagen through cytokine secretion, impairing left ventricular ejection fraction. 4) As central immunoregulators in innate and adaptive immunity, dendritic cells (DCs) play crucial roles in promoting wound healing after myocardial infarction (MI). DCs can inhibit inflammation, thereby suppressing myocardial fibrosis and enhancing cardiac function.

The lymphatic system, an essential part of the body’s immune defense, is intricately connected with blood vessels, forming a complex network that plays roles in interstitial fluid drainage, lipid absorption, and immune cell responses (25). The relationship between cardiac lymphatics and immune cells is mutually influential; cardiac lymphatics provide pathways for immune cell and inflammatory cytokine clearance, while immune cells promote or inhibit cardiac lymphatic endothelial cell growth and survival, affecting lymphatic structure and function (72) (**Figure 4**).

#### 6.2.1 Neutrophil and cardiac lymphatics

The heart possesses an extensive lymphatic network. With each cardiac contraction, extracellular fluid and wastes in the myocardium are expelled and conveyed to the draining lymph nodes around the aortic arch. During inflammation, infiltrating neutrophils release enzymes (such as neutrophil elastase MPO) to break down Emilin-1anchoring filaments, reducing their attachment to the extracellular matrix and causing the lymphatic vessels to collapse. This impairs lymphatic transport and exacerbates myocardial edema (73). Studies have shown that upregulating LYVE-1 can stimulate cardiac lymphangiogenesis and effectively transport inflammatory cells to the mediastinal lymph nodes. This improves the clearance rate of acute inflammatory responses following myocardial infarction, promoting myocardial tissue repair and improving cardiac function. At the same time, LYVE-1 deficiency can inhibit neutrophil translocation through lymphatic endothelium and exacerbate chronic inflammation and long-term deterioration of cardiac function (31).

#### 6.2.2 Macrophages and cardiac lymphatics

Macrophages, as the main immune cells involved in the inflammatory response to MI, are key effectors of inflammation and innate immunity, and the immune-inflammatory response they mediate is involved in the whole process of “damage-repair” of cardiac tissues after MI (74). M2b macrophages, also known as regulatory macrophages, can repair the heart by directly inhibiting the overproduction of proinflammatory cytokines by M1-type macrophages. At the same time, the production of vascular endothelial growth factor-C (VEGF-C) plays an important role in cardiac lymphatic neovascularization and regulation of the immune microenvironment (75). Wang et al. (76) found in an *in vitro* LECs model that M2b macrophage cultures could upregulate vascular endothelial growth factor receptor-3 (VEGFR-3) and VEGF-C expression in LECs, stimulate cell migration and cell proliferation of LECs, and increase the total length of the lymphatic vessels, the length of the total branching, the number of the nodes and the number of the nodal points; In an *in vivo* MI/RI rat model, cardiac M2b macrophage transplantation upregulated VEGFR-3 and VEGF-C expression, promoted lymphatic vessel neogenesis, inhibited myocardial fibrosis, and improved cardiac function. Patricia et al. (77) found that cardiac macrophage efferocytosis induces VEGFC release, and VEGF-C functions in a paracrine fashion to the promotion of myocardial lymphangiogenesis and modulate the presence of immune cells, such as myeloid cells expressing low levels of MHCII and Tregs as well as in an autocrine manner to dampen macrophage proinflammatory cytokine production, thereby resolving inflammation and promoting myocardial repair. In addition, VEGF-C production by cardiac macrophages through cellular burial can directly inhibit macrophage over-secretion of proinflammatory cytokines to ameliorate myocardial injury and promote cardiac healing, in addition to promoting cardiac lymphangiogenesis and performing antigen clearance (78). Another study reported that the suppression of PFKFB3 expression in macrophages could enhance the generation of VEGF-C in macrophages and promote lymphatic neogenesis and the mitigation of the inflammatory response following MI/RI.

#### 6.2.3 Cardiac lymphatics and lymphocytes

B lymphocytes are one of the most abundant immune cells in the organism. B lymphocytes provide antigens to T cells and produce cytokines and chemokines, which ultimately regulate the function of other immune cells. T cells are a significant population of cells involved in cellular immunity and play an essential role in the repair and regeneration of myocardial injuries. T cells negatively regulate the production of lymphatic vessels, in which the type 1 helper T cells secrete a polytropic cytokine, γ-interferon, which plays a vital role in initiating cell-mediated adaptive immune responses as well as regulating cell growth and differentiation (79). Houssari et al (72) investigated the role of cardiac recruitment of T cells in lymphatic remodeling after myocardial infarction. They found that treatment with the VEGF-C/VEGF-D trap (soluble VEGFR3) restricted the recruitment of T cells into the infarcted area, resulting in reduced left ventricular wall thinning, delayed scar remodeling, and reduced cardiac dysfunction after myocardial infarction. Further studies confirmed the deleterious effects of cardiac infiltrating T cells (including CD4^+^ and CD8^+^ subpopulations) on the cardiac lymphatic system after myocardial infarction. They inhibited cardiac lymphovascular neogenesis in the acute phase after myocardial infarction to a certain extent via IFN-γ. Mature B lymphocytes alter the recovery of cardiac function after AMI in mice (80). It has been shown that activated B lymphocytes are involved in the persistence of myocardial inflammation and immune system activation after myocardial infarction. They may impair left ventricular ejection function by secreting cytokines to promote the expression of myocardial type I and type III collagen (81).

#### 6.2.4 Cardiac lymphatics and dendritic cells

As the central immunoregulators in innate and adaptive immunity, DCs are crucial in promoting wound healing after MI (37). Research reported that Mice depleted of DCs could cause a severe inflammatory response and worse left ventricular remodeling after MI (62).I In addition, exosomes derived from DCs could migrate to lymphoid tissue and improve cardiac function after myocardial infarction via activation of CD4^+^ T cells (82). Dendritic cells are important cells involved in regulating lymphatic return (83). In the inflammatory state, increased vascular permeability and transmural blood flow enhance the expression and secretion of CCL21 by lymphatic vessel endothelial cells, which promotes the delivery of dendritic cells to lymph nodes (84). Within the lumen of lymphatic capillaries, DCs crawl towards collecting vessels by CCL21 chemokine gradients before falling into collecting vessels where cells are passively transported by lymph flow (85). Increased vascular permeability leads to fluid and protein influx into the intercellular matrix, elevated interstitial fluid pressure, and compensatory increase in lymphatic return (84, 86). Another study showed that routine conditioned cultures of lymphatic endothelial cells significantly inhibited the maturation of dendritic cells, which may be a novel mechanism by which lymphatic endothelial cells regulate cellular immune responses and limit inflammation (87). furthermore, DCs can lymphoangiogenesis during inflammation and regulate lymphatic vessel integrity (85).

## 7 Treatment strategies for AMI

### 7.1 Regulation of immune inflammation

MI is closely related to the body’s immune-inflammatory response. Acute myocardial ischemic episodes induce cellular injury and death of cardiomyocytes, endothelial cells, fibroblasts, and other cells, which initiate an acute pro-inflammatory response through the synergistic action of several processes: complement cascade activation, reactive oxygen species production, and damage-related molecular patterns (88). Neutrophils are the forerunners of the inflammatory response. In addition, a variety of immune cells (e.g., macrophages, dendritic cells, lymphocytes, eosinophils, etc.) are recruited to the damaged area through multiple pathways to remove dead cardiomyocytes and stromal remnants, secrete cytokines and growth factors, promote fibroblast activation and proliferation, and regulate the wound healing response and ventricular remodeling after myocardial infarction (40). It has been found that AMI is often accompanied by immune dysfunction, and immune dysfunction can also directly or indirectly cause the expansion of MI area or complications (72). The immune system plays a vital role in the post-MI process. Once an ischemic injury occurs, a robust inflammatory cell infiltration is initiated in the heart to remove the dead tissue (89). A growing body of evidence suggests that timely resolution of the inflammatory process may aid in preventing adverse cardiac remodeling and HF (90). However, excessive or persistent inflammation can lead to adverse left ventricle remodeling and the development of HF (91). The promising therapeutic strategy of anti-inflammatory and immunomodulatory therapies in MI patients during acute and chronic cardiac injury phases (89).

### 7.2 Promote the regeneration of cardiac lymphatics

Selective stimulation of cardiac lymphangiogenesis can help regulate the myocardial inflammatory microenvironment after MI, promote myocardial survival and recovery, improve myocardial fluid balance, and reduce the occurrence of myocardial edema and fibrosis after injury (14). One study reported that increased cardiac inflammation and edema were found by blocking endogenous VEGF-C to inhibit lymphangiogenesis. At the same time, VEGF-C treatment significantly reduced inflammation and edema and improved cardiac function, suggesting that modulation of endogenous lymphatic neogenesis has a potential therapeutic role in myocardial injury (14). A study on myocardial ischemia model mice, through the inhibition of VEGF-C and VEGFR3 (92), found that the suppression of endogenous lymphangiogenesis led to an increase in infarct scar size, ventricular hypertrophy left ventricular dilatation, and a decrease in left ventricular function. In contrast, selective stimulation of cardiac lymphangiogenesis, improved remodeling of collecting and collecting lymphatics, and accelerated cardiac lymphatic transport capacity were sufficient to attenuate myocardial edema, inflammation, and fibrosis after myocardial infarction in rats, thereby reducing pathologic remodeling, improving cardiac function, and partially preventing the development of HF. In addition, cytokines secreted by LEC can promote cardiomyocyte proliferation and survival, reduce cardiomyocyte apoptosis, and protect the heart after myocardial infarction (93). Hartikainen et al. (94), in their study on the mouse infarction model, found that sustained VEGF-CC156 S treatment via adeno-associated viral vectors effectively increased cardiac lymphangiogenesis and reduced cardiac inflammation and dysfunction at 3 weeks after myocardial infarction. Cardiac inflammation and dysfunction 3 weeks after myocardial infarction. In recent years, many studies have been done to improve lymphatic drainage in infarcted myocardium by stimulating lymphatic vessel neogenesis. Wang et al. (95) observed an increase in lymphatic vessels in the infarcted and infarct border areas after epicardial transplantation of patches bearing bone marrow-derived mesenchymal stem cells. Trincot et al. (96) found that adrenomedullin produced by epicardium regulated lymphatic vessel neogenesis after myocardial infarction and that over-expression of adrenomedullin was associated with increased lymphatic vessel neogenesis after myocardial infarction. Expression of adrenomedullin enhanced lymphatic vessel neogenesis, reduced cardiac edema, and improved cardiac function. Klotz et al. (69) observed enhanced lymphovascular neogenesis in myocardial injury and significant improvement in cardiac function after intraperitoneal injection of VEGF-C in mice. Henri et al. (14) delivered VEGF-C with nanoparticles prepared from albumin and alginate, which alleviated lymphovascular conformational changes, reduced cardiac edema and inflammatory response, and enhanced cardiac function after intramyocardial injection in rats. Vieiraet al (31) reduced the acute inflammatory response after myocardial infarction by inducing lymphovascular transport of immune cells to mediastinal lymph nodes via intraperitoneal injection of VEGF-C in mice. Lymphangiogenesis is an essential step in the process of myocardial repair. In addition to its potential therapeutic value, the role of lymphangiogenesis in providing a suitable microenvironment for myocardial regeneration should not be underestimated, and lymphangiogenesis will likely be a breakthrough in future myocardial regeneration research.

### 7.3 Improve lymphatic drainage function

Lymphatic vessels are involved in fluid transport and play an important role in fluid homeostasis. When lymphatic vessels are insufficiently neoplastic or maladaptive, it leads to lymphatic transport dysfunction, leading to chronic interstitial edema and inflammatory response and triggering interstitial fibrosis. Animal experiments have shown that insufficient lymphatic drainage worsens myocardial edema and inflammation and aggravates myocardial injury (68). In a rat model of MI caused by occlusion of the left anterior descending branch of the coronary artery, the lymphatics in the infarcted area were almost absent. However, the lymphatics in the non-infarcted area of the left ventricle were extensively remodeled.

Meanwhile, although myocardial infarction significantly induced initial lymphangiogenesis in the heart, poor remodeling of subepicardial pre-collecting and collecting lymphatics resulted in decreased cardiac lymphatic transport capacity, which was insufficient to maintain cardiac fluid homeostasis, thus promoting the development of chronic myocardial edema and inflammation and exacerbating myocardial fibrosis and cardiac dysfunction, whereas, after VEGF treatment, the subepicardial pre-collecting lymphatics in infarcted borderline regions and the collecting lymphatic vessels were partially improved. Interstitial myocardial edema was significantly reduced (14). The above studies suggest that despite the endogenous lymphangiogenic response after myocardial infarction, remodeling and dysfunction of collecting duct lymph can still lead to a decrease in cardiac lymphatic transport capacity, leading to chronic myocardial edema and inflammation, aggravating myocardial fibrosis and causing cardiac dysfunction. Therefore, improving lymphatic return function can reduce interstitial edema and effectively discharge tissue metabolites after hypoxia, thus improving post-infarction cardiac function. In addition, during the formation of granulation tissue after myocardial infarction, lymphatics participate in the maturation of fibrosis and the formation of scar tissue by draining excess proteins and fluids (67), providing a suitable microenvironment for possible myocardial regeneration. Early studies of lymphatic vessel ligation-induced chronic myocardial edema in dogs showed that lymphatic vessel dysfunction leads to endocardial myocardial fibrosis and endocardial fibroelastic degeneration.

## 8 Conclusion and perspectives

Lymphatic vessels and the immune system interact and influence each other, and both play important roles in two pathological stages: early inflammatory injury and late ventricular remodeling after MI. Lymphatic vessels act as channels for immune cell drainage and participate in the immune cell-mediated inflammatory response after MI. Relevant studies have demonstrated that enhanced cardiac lymphangiogenesis can accelerate the removal of infiltrating immune cells, reduce the production of pro-inflammatory cytokines, attenuate myocardial edema, inflammatory response, and myocardial fibrosis, and promote the recovery of damaged cardiac function (14). At the same time, immune cells promote or inhibit the growth and survival of cardiac lymphatic endothelial cells, affecting the structure and function of cardiac lymphatic vessels. After myocardial infarction, the cardiac lymphatic vascular system coordinates the immune response and cardiac remodeling process, especially macrophages, which regulate the immune response and cardiac repair after MI through lymphatic transport in the damaged heart. Inadequate or dysfunctional lymphangiogenesis plays a vital role in myocardial remodeling and cardiac dysfunction in MI and nonischemic heart disease. Targeted immunomodulation through modulation of lymphangiogenesis and lymphatic endothelial cell secretion is of great clinical value for treating myocardial injury.

Currently, questions regarding the response and function of cardiac lymphatics in the disease environment need to be further investigated and translated to provide effective therapeutic strategies so that targeted modulation of cardiac lymphangiogenesis to improve fluid homeostasis, modulation of the immune response, and downstream fibrosis can be an effective strategy to promote cardiac repair. The lymphatic system’s immune cells and immune factors are involved in and regulate myocardial injury, neovascularization, and ventricular remodeling after myocardial infarction at multiple levels. Lymphatic vessel neovascularization and pooled lymphatic vessel remodeling are crucial to ensure lymphatic return and prevent excessive inflammatory response; therefore, targeting the lymphatic system/immune cell axis may provide a new research direction for comprehensive treatment after myocardial infarction. Further study is needed better to integrate lymphatic drainage and immune response after myocardial infarction.

## References

[1] Duque-Ossa LC Garcia-Ferrera B Reyes-Retana JA. Troponin I as a biomarker for early detection of acute myocardial infarction. Curr Probl Cardio. (2023) 48:101067. doi: 10.1016/j.cpcardiol.2021.101067 34826431

[2] Krittanawong C Khawaja M Tamis-Holland JE Girotra S Rao SV. Acute myocardial infarction: etiologies and mimickers in young patients. J Am Heart Asso. (2023) 12:e029971. doi: 10.1161/JAHA.123.029971 PMC10547302 37724944

[3] Zhou W Li K Wei Y Hao P Chi M Liu Y. Ultrasensitive label-free optical microfiber coupler biosensor for detection of cardiac troponin I based on interference turning point effect. Biosens Bioelectron. (2018) 106:99–104. doi: 10.1016/j.bios.2018.01.061 29414096

[4] Samsky MD Morrow DA Proudfoot AG Hochman JS Thiele H Rao SV. Cardiogenic shock after acute myocardial infarction: A review. JAMA. (2021) 326:1840–50. doi: 10.1001/jama.2021.18323 PMC9661446 34751704

[5] Sterling LH Fernando SM Talarico R Qureshi D van Diepen S Herridge MS. Long-term outcomes of cardiogenic shock complicating myocardial infarction. J Am Coll Cardio. (2023) 82:985–95. doi: 10.1016/j.jacc.2023.06.026 37648357

[6] Choo EH Lee JH Park EH Park HE Jung NC Kim TH. Infarcted myocardium-primed dendritic cells improve remodeling and cardiac function after myocardial infarction by modulating the regulatory T cell and macrophage polarization. Circulation. (2017) 135:1444–57. doi: 10.1161/CIRCULATIONAHA.116.023106 28174192

[7] Jiang H Fang T Cheng Z. Mechanism of heart failure after myocardial infarction. J Int Med Re. (2023) 51:3000605231202573. doi: 10.1177/03000605231202573 PMC10566288 37818767

[8] Lv J Deng C Jiang S Ji T Yang Z Wang Z. Blossoming 20: the energetic regulator’s birthday unveils its versatility in cardiac diseases. Theranostics. (2019) 9:466–76. doi: 10.7150/thno.29130 PMC6376194 30809287

[9] Matter MA Paneni F Libby P Frantz S Stahli BE Templin C. Inflammation in acute myocardial infarction: the good, the bad and the ugly. Eur Heart. (2024) 45:89–103. doi: 10.1093/eurheartj/ehad486 PMC10771378 37587550

[10] Brakenhielm E Gonzalez A Diez J. Role of cardiac lymphatics in myocardial edema and fibrosis: JACC review topic of the week. J Am Coll Cardio. (2020) 76:735–44. doi: 10.1016/j.jacc.2020.05.076 32762908

[11] Klaourakis K Vieira JM Riley PR. The evolving cardiac lymphatic vasculature in development, repair and regeneration. Nat Rev Cardio. (2021) 18:368–79. doi: 10.1038/s41569-020-00489-x PMC7812989 33462421

[12] Angeli V Lim HY. Biomechanical control of lymphatic vessel physiology and functions. Cell Mol Immuno. (2023) 20:1051–62. doi: 10.1038/s41423-023-01042-9 PMC10469203 37264249

[13] Heron C Dumesnil A Houssari M Renet S Lemarcis T Lebon A. Regulation and impact of cardiac lymphangiogenesis in pressure-overload-induced heart failure. Cardiovasc Re. (2023) 119:492–505. doi: 10.1093/cvr/cvac086 PMC10064842 35689481

[14] Henri O Pouehe C Houssari M Galas L Nicol L Edwards-Levy F. Selective stimulation of cardiac lymphangiogenesis reduces myocardial edema and fibrosis leading to improved cardiac function following myocardial infarction. Circulation. (2016) 133:1484–1497;discussion 1497. doi: 10.1161/CIRCULATIONAHA.115.020143 26933083

[15] Petrova TV Koh GY. Biological functions of lymphatic vessels. Science. (2020) 369:eaax4063. doi: 10.1126/science.aax4063 32646971

[16] Yang Y Oliver G. Development of the mammalian lymphatic vasculature. J Clin Invest. (2014) 124:888–97. doi: 10.1172/JCI71609 PMC3938267 24590273

[17] Venero Galanternik M Stratman AN Jung HM Butler MG Weinstein BM. Building the drains: the lymphatic vasculature in health and disease. Wiley Interdiscip Rev Dev Bio. (2016) 5:689–710. doi: 10.1002/wdev.2016.5.issue-6 27576003

[18] Ma W Oliver G. Lymphatic endothelial cell plasticity in development and disease. Physiol (Bethesda). (2017) 32:444–52. doi: 10.1152/physiol.00015.2017 PMC5817163 29021364

[19] Pichol-Thievend C Betterman KL Liu X Ma W Skoczylas R Lesieur E. A blood capillary plexus-derived population of progenitor cells contributes to genesis of the dermal lymphatic vasculature during embryonic development. Development. (2018) 145:dev160184. doi: 10.1242/dev.160184 29773646 PMC6001371

[20] Yazdani S Navis G Hillebrands JL van Goor H van den Born J. Lymphangiogenesis in renal diseases: passive bystander or active participant? Expert Rev Mol Me. (2014) 16:e15. doi: 10.1017/erm.2014.18 25252809

[21] Wiig H Swartz MA. Interstitial fluid and lymph formation and transport: physiological regulation and roles in inflammation and cancer. Physiol Re. (2012) 92:1005–60. doi: 10.1152/physrev.00037.2011 22811424

[22] Trzewik J Mallipattu SK Artmann GM Delano FA Schmid-Schonbein GW. Evidence for a second valve system in lymphatics: endothelial microvalves. FASEB. (2001) 15:1711–7. doi: 10.1096/fj.01-0067com 11481218

[23] von der Weid PY Zawieja DC. Lymphatic smooth muscle: the motor unit of lymph drainage. Int J Biochem Cell Bio. (2004) 36:1147–53. doi: 10.1016/j.biocel.2003.12.008 15109561

[24] Van Helden DF. Pacemaker potentials in lymphatic smooth muscle of the Guinea-pig mesentery. J Physio. (1993) 471:465–79. doi: 10.1113/jphysiol.1993.sp019910 PMC1143971 8120817

[25] Morfoisse F Noel A. Lymphatic and blood systems: Identical or fraternal twins? Int J Biochem Cell Bio. (2019) 114:105562. doi: 10.1016/j.biocel.2019.105562 31278994

[26] Ulvmar MH Makinen T. Heterogeneity in the lymphatic vascular system and its origin. Cardiovasc Re. (2016) 111:310–21. doi: 10.1093/cvr/cvw175 PMC4996263 27357637

[27] Zheng Z Ren K Peng X Zhu X Yi G. Lymphatic vessels: A potential approach to the treatment of atherosclerosis? Lymphat Res Bio. (2018) 16:498–506. doi: 10.1089/lrb.2018.0015 30272526

[28] Brouillard P Boon L Vikkula M. Genetics of lymphatic anomalies. J Clin Invest. (2014) 124:898–904. doi: 10.1172/JCI71614 24590274 PMC3938256

[29] Hara H Mihara M Ohtomo R Tanaka S. Lymphatic vessel thrombosis in a patient with secondary lymphedema. Plast Reconstr Surg Glob Ope. (2019) 7:e2268. doi: 10.1097/GOX.0000000000002268 PMC6571332 31333981

[30] Liu X Oliver G. The lymphatic vasculature in cardiac development and ischemic heart disease. Circ Re. (2023) 132:1246–53. doi: 10.1161/CIRCRESAHA.122.321672 PMC10154041 37104562

[31] Vieira JM Norman S Villa Del Campo C Cahill TJ Barnette DN Gunadasa-Rohling M. The cardiac lymphatic system stimulates resolution of inflammation following myocardial infarction. J Clin Invest. (2018) 128:3402–12. doi: 10.1172/JCI97192 PMC6063482 29985167

[32] Berkeley B Tang MNH Brittan M. Mechanisms regulating vascular and lymphatic regeneration in the heart after myocardial infarction. J Patho. (2023) 260:666–78. doi: 10.1002/path.v260.5 PMC10953458 37272582

[33] Frangogiannis NG. The inflammatory response in myocardial injury, repair, and remodelling. Nat Rev Cardio. (2014) 11:255–65. doi: 10.1038/nrcardio.2014.28 PMC4407144 24663091

[34] You H Dong M. Identification of immuno-inflammation-related biomarkers for acute myocardial infarction based on bioinformatics. J Inflammation Re. (2023) 16:3283–302. doi: 10.2147/JIR.S421196 PMC10417757 37576155

[35] Cai S Zhao M Zhou B Yoshii A Bugg D Villet O. Mitochondrial dysfunction in macrophages promotes inflammation and suppresses repair after myocardial infarction. J Clin Invest. (2023) 133:e159498. doi: 10.1172/JCI159498 36480284 PMC9927948

[36] Andreadou I Cabrera-Fuentes HA Devaux Y Frangogiannis NG Frantz S Guzik T. Immune cells as targets for cardioprotection: new players and novel therapeutic opportunities. Cardiovasc Re. (2019) 115:1117–30. doi: 10.1093/cvr/cvz050 PMC6529904 30825305

[37] Sun K Li YY Jin J. A double-edged sword of immuno-microenvironment in cardiac homeostasis and injury repair. Signal Transduct Target The. (2021) 6:79. doi: 10.1038/s41392-020-00455-6 PMC7897720 33612829

[38] Francis Stuart SD De Jesus NM Lindsey ML Ripplinger CM. The crossroads of inflammation, fibrosis, and arrhythmia following myocardial infarction. J Mol Cell Cardio. (2016) 91:114–22. doi: 10.1016/j.yjmcc.2015.12.024 PMC4764395 26739214

[39] DeBerge M Yu S Dehn S Ifergan I Yeap XY Filipp M. Monocytes prime autoreactive T cells after myocardial infarction. Am J Physiol Heart Circ Physio. (2020) 318:H116–23. doi: 10.1152/ajpheart.00595.2019 PMC6985803 31809213

[40] Kologrivova I Shtatolkina M Suslova T Ryabov V. Cells of the immune system in cardiac remodeling: main players in resolution of inflammation and repair after myocardial infarction. Front Immuno. (2021) 12:664457. doi: 10.3389/fimmu.2021.664457 PMC8050340 33868315

[41] Silvis MJM Kaffka Genaamd Dengler SE Odille CA Mishra M van der Kaaij NP Doevendans PA. Damage-associated molecular patterns in myocardial infarction and heart transplantation: the road to translational success. Front Immuno. (2020) 11:599511. doi: 10.3389/fimmu.2020.599511 PMC7752942 33363540

[42] Daseke MJ 2nd Chalise U Becirovic-Agic M Salomon JD Cook LM Case AJ. Neutrophil signaling during myocardial infarction wound repair. Cell Signa. (2021) 77:109816. doi: 10.1016/j.cellsig.2020.109816 PMC7718402 33122000

[43] Puhl SL Steffens S. Neutrophils in post-myocardial infarction inflammation: damage vs. Resolution? Front Cardiovasc Me. (2019) 6:25. doi: 10.3389/fcvm.2019.00025 PMC6431642 30937305

[44] Thiam HR Wong SL Wagner DD Waterman CM. Cellular mechanisms of NETosis. Annu Rev Cell Dev Bio. (2020) 36:191–218. doi: 10.1146/annurev-cellbio-020520-111016 32663035 PMC8499668

[45] Sorvillo N Cherpokova D Martinod K Wagner DD. Extracellular DNA NET-works with dire consequences for health. Circ Re. (2019) 125:470–88. doi: 10.1161/CIRCRESAHA.119.314581 PMC6746252 31518165

[46] Helseth R Shetelig C Andersen GO Langseth MS Limalanathan S Opstad TB. Neutrophil extracellular trap components associate with infarct size, ventricular function, and clinical outcome in STEMI. Mediators Inflam. (2019) 2019:7816491. doi: 10.1155/2019/7816491 PMC6854936 31772506

[47] Du M Yang W Schmull S Gu J Xue S. Inhibition of peptidyl arginine deiminase-4 protects against myocardial infarction induced cardiac dysfunction. Int Immunopharmacol. (2020) 78:106055. doi: 10.1016/j.intimp.2019.106055 31816575

[48] Vogel B Shinagawa H Hofmann U Ertl G Frantz S. Acute DNase1 treatment improves left ventricular remodeling after myocardial infarction by disruption of free chromatin. Basic Res Cardio. (2015) 110:15. doi: 10.1007/s00395-015-0472-y 25702039

[49] Chen C Zhang H Xie R Wang Y Ma Y. Gut microbiota aggravate cardiac ischemia-reperfusion injury via regulating the formation of neutrophils extracellular traps. Life Sc. (2022) 303:120670. doi: 10.1016/j.lfs.2022.120670 35640777

[50] Li YW Chen SX Yang Y Zhang ZH Zhou WB Huang YN. Colchicine inhibits NETs and alleviates cardiac remodeling after acute myocardial infarction. Cardiovasc Drugs The. (2024) 38:31–41. doi: 10.1007/s10557-022-07326-y 35900652

[51] Lemarie J Boufenzer A Popovic B Tran N Groubatch F Derive M. Pharmacological inhibition of the triggering receptor expressed on myeloid cells-1 limits reperfusion injury in a porcine model of myocardial infarction. ESC Heart Fai. (2015) 2:90–9. doi: 10.1002/ehf2.12029 PMC6410538 28834656

[52] Hilgendorf I Gerhardt LM Tan TC Winter C Holderried TA Chousterman BG. Ly-6Chigh monocytes depend on Nr4a1 to balance both inflammatory and reparative phases in the infarcted myocardium. Circ Re. (2014) 114:1611–22. doi: 10.1161/CIRCRESAHA.114.303204 PMC4017349 24625784

[53] Peet C Ivetic A Bromage DI Shah AM. Cardiac monocytes and macrophages after myocardial infarction. Cardiovasc Re. (2020) 116:1101–12. doi: 10.1093/cvr/cvz336 PMC7177720 31841135

[54] Pavlou S Lindsay J Ingram R Xu H Chen M. Sustained high glucose exposure sensitizes macrophage responses to cytokine stimuli but reduces their phagocytic activity. BMC Immuno. (2018) 19:24. doi: 10.1186/s12865-018-0261-0 PMC6042333 29996768

[55] Kim Y Nurakhayev S Nurkesh A Zharkinbekov Z Saparov A. Macrophage polarization in cardiac tissue repair following myocardial infarction. Int J Mol Sc. (2021) 22:2715. doi: 10.3390/ijms22052715 33800220 PMC7962533

[56] Tang Y Lin X Chen C Tong Z Sun H Li Y. Nucleolin improves heart function during recovery from myocardial infarction by modulating macrophage polarization. J Cardiovasc Pharmacol The. (2021) 26:386–95. doi: 10.1177/1074248421989570 33550832

[57] Weirather J Hofmann UD Beyersdorf N Ramos GC Vogel B Frey A. Foxp3+ CD4+ T cells improve healing after myocardial infarction by modulating monocyte/macrophage differentiation. Circ Re. (2014) 115:55–67. doi: 10.1161/CIRCRESAHA.115.303895 24786398

[58] Saxena A Dobaczewski M Rai V Haque Z Chen W Li N. Regulatory T cells are recruited in the infarcted mouse myocardium and may modulate fibroblast phenotype and function. Am J Physiol Heart Circ Physio. (2014) 307:H1233–1242. doi: 10.1152/ajpheart.00328.2014 PMC4200341 25128167

[59] Yan X Anzai A Katsumata Y Matsuhashi T Ito K Endo J. Temporal dynamics of cardiac immune cell accumulation following acute myocardial infarction. J Mol Cell Cardio. (2013) 62:24–35. doi: 10.1016/j.yjmcc.2013.04.023 23644221

[60] Zouggari Y Ait-Oufella H Bonnin P Simon T Sage AP Guerin C. B lymphocytes trigger monocyte mobilization and impair heart function after acute myocardial infarction. Nat Me. (2013) 19:1273–80. doi: 10.1038/nm.3284 PMC4042928 24037091

[61] Worbs T Hammerschmidt SI Forster R. Dendritic cell migration in health and disease. Nat Rev Immuno. (2017) 17:30–48. doi: 10.1038/nri.2016.116 27890914

[62] Anzai A Anzai T Nagai S Maekawa Y Naito K Kaneko H. Regulatory role of dendritic cells in postinfarction healing and left ventricular remodeling. Circulation. (2012) 125:1234–45. doi: 10.1161/CIRCULATIONAHA.111.052126 22308302

[63] Nagai T Honda S Sugano Y Matsuyama TA Ohta-Ogo K Asaumi Y. Decreased myocardial dendritic cells is associated with impaired reparative fibrosis and development of cardiac rupture after myocardial infarction in humans. J Am Heart Assoc. (2014) 3:e000839. doi: 10.1161/JAHA.114.000839 24895162 PMC4309075

[64] Henein MY Vancheri S Longo G Vancheri F. The role of inflammation in cardiovascular disease. Int J Mol Sc. (2022) 23:12906. doi: 10.3390/ijms232112906 36361701 PMC9658900

[65] Nakkala JR Yao Y Zhai Z Duan Y Zhang D Mao Z. Dimethyl itaconate-loaded nanofibers rewrite macrophage polarization, reduce inflammation, and enhance repair of myocardic infarction. Small. (2021) 17:e2006992. doi: 10.1002/smll.202006992 33719217

[66] Ravaud C Ved N Jackson DG Vieira JM Riley PR. Lymphatic clearance of immune cells in cardiovascular disease. Cells. (2021) 10:2594. doi: 10.3390/cells10102594 34685572 PMC8533855

[67] Ishikawa Y Akishima-Fukasawa Y Ito K Akasaka Y Tanaka M Shimokawa R. Lymphangiogenesis in myocardial remodelling after infarction. Histopathology. (2007) 51:345–53. doi: 10.1111/j.1365-2559.2007.02785.x PMC2366023 17727476

[68] Bai J Yin L Yu WJ Zhang YL Lin QY Li HH. Angiotensin II induces cardiac edema and hypertrophic remodeling through lymphatic-dependent mechanisms. Oxid Med Cell Longe. (2022) 2022:5044046. doi: 10.1155/2022/5044046 PMC8881141 35222798

[69] Klotz L Norman S Vieira JM Masters M Rohling M Dube KN. Cardiac lymphatics are heterogeneous in origin and respond to injury. Nature. (2015) 522:62–7. doi: 10.1038/nature14483 PMC4458138 25992544

[70] Ge Z Yin C Li Y Tian D Xiang Y Li Q. Long noncoding RNA NEAT1 promotes cardiac fibrosis in heart failure through increased recruitment of EZH2 to the Smad7 promoter region. J Transl Me. (2022) 20:7. doi: 10.1186/s12967-021-03211-8 PMC8722118 34980170

[71] Verma S Mazer CD Yan AT Mason T Garg V Teoh H. Effect of empagliflozin on left ventricular mass in patients with type 2 diabetes mellitus and coronary artery disease: the EMPA-HEART cardioLink-6 randomized clinical trial. Circulation. (2019) 140:1693–702. doi: 10.1161/CIRCULATIONAHA.119.042375 31434508

[72] Houssari M Dumesnil A Tardif V Kivela R Pizzinat N Boukhalfa I. Lymphatic and immune cell cross-talk regulates cardiac recovery after experimental myocardial infarction. Arterioscler Thromb Vasc Bio. (2020) 40:1722–37. doi: 10.1161/ATVBAHA.120.314370 PMC7310303 32404007

[73] Janssen EM Dy SM Meara AS Kneuertz PJ Presley CJ Bridges JFP. Analysis of patient preferences in lung cancer - estimating acceptable tradeoffs between treatment benefit and side effects. Patient Prefer Adherence. (2020) 14:927–37. doi: 10.2147/PPA.S235430 PMC7276327 32581519

[74] Zuo W Sun R Ji Z Ma G. Macrophage-driven cardiac inflammation and healing: insights from homeostasis and myocardial infarction. Cell Mol Biol Let. (2023) 28:81. doi: 10.1186/s11658-023-00491-4 37858035 PMC10585879

[75] Glinton KE Ma W Lantz C Grigoryeva LS DeBerge M Liu X. Macrophage-produced VEGFC is induced by efferocytosis to ameliorate cardiac injury and inflammation. J Clin Invest. (2022) 132:e140685. doi: 10.1172/JCI140685 35271504 PMC9057589

[76] Wang C Yue Y Huang S Wang K Yang X Chen J. M2b macrophages stimulate lymphangiogenesis to reduce myocardial fibrosis after myocardial ischaemia/reperfusion injury. Pharm Bio. (2022) 60:384–93. doi: 10.1080/13880209.2022.2033798 PMC8865132 35188856

[77] D’Amore PA Alcaide P. Macrophage efferocytosis with VEGFC and lymphangiogenesis: rescuing the broken heart. J Clin Invest. (2022) 132:e158703. doi: 10.1172/JCI158703 35499075 PMC9057620

[78] Tammela T Alitalo K. Lymphangiogenesis: Molecular mechanisms and future promise. Cell. (2010) 140:460–76. doi: 10.1016/j.cell.2010.01.045 20178740

[79] Varma TK Lin CY Toliver-Kinsky TE Sherwood ER. Endotoxin-induced gamma interferon production: contributing cell types and key regulatory factors. Clin Diagn Lab Immuno. (2002) 9:530–43. doi: 10.1128/CDLI.9.3.530-543.2002 PMC119981 11986256

[80] Sun Y Pinto C Camus S Duval V Alayrac P Zlatanova I. Splenic marginal zone B lymphocytes regulate cardiac remodeling after acute myocardial infarction in mice. J Am Coll Cardio. (2022) 79:632–47. doi: 10.1016/j.jacc.2021.11.051 35177192

[81] Mo F Luo Y Yan Y Li J Lai S Wu W. Are activated B cells involved in the process of myocardial fibrosis after acute myocardial infarction? An *in vivo* experiment. BMC Cardiovasc Disor. (2021) 21:5. doi: 10.1186/s12872-020-01775-9 PMC7789158 33407160

[82] Liu H Gao W Yuan J Wu C Yao K Zhang L. Exosomes derived from dendritic cells improve cardiac function via activation of CD4(+) T lymphocytes after myocardial infarction. J Mol Cell Cardio. (2016) 91:123–33. doi: 10.1016/j.yjmcc.2015.12.028 26746143

[83] Christiansen AJ Dieterich LC Ohs I Bachmann SB Bianchi R Proulx ST. Lymphatic endothelial cells attenuate inflammation via suppression of dendritic cell maturation. Oncotarget. (2016) 7:39421–35. doi: 10.18632/oncotarget.v7i26 PMC5129942 27270646

[84] Miteva DO Rutkowski JM Dixon JB Kilarski W Shields JD Swartz MA. Transmural flow modulates cell and fluid transport functions of lymphatic endothelium. Circ Re. (2010) 106:920–31. doi: 10.1161/CIRCRESAHA.109.207274 PMC10994404 20133901

[85] Permanyer M Bosnjak B Forster R. Dendritic cells, T cells and lymphatics: dialogues in migration and beyond. Curr Opin Immuno. (2018) 53:173–9. doi: 10.1016/j.coi.2018.05.004 29857205

[86] He C Young AJ West CA Su M Konerding MA Mentzer SJ. Stimulation of regional lymphatic and blood flow by epicutaneous oxazolone. J Appl Physiol (1985). (2002) 93:966–73. doi: 10.1152/japplphysiol.00212.2002 12183492

[87] Norman S Riley PR. Anatomy and development of the cardiac lymphatic vasculature: Its role in injury and disease. Clin Anat. (2016) 29:305–15. doi: 10.1002/ca.22638 26443964

[88] Ong SB Hernandez-Resendiz S Crespo-Avilan GE Mukhametshina RT Kwek XY Cabrera-Fuentes HA. Inflammation following acute myocardial infarction: Multiple players, dynamic roles, and novel therapeutic opportunities. Pharmacol The. (2018) 186:73–87. doi: 10.1016/j.pharmthera.2018.01.001 PMC5981007 29330085

[89] Viola M de Jager SCA Sluijter JPG. Targeting inflammation after myocardial infarction: A therapeutic opportunity for extracellular vesicles? Int J Mol Sc. (2021) 22:7831. doi: 10.3390/ijms22157831 34360595 PMC8346058

[90] Frangogiannis NG. Regulation of the inflammatory response in cardiac repair. Circ Re. (2012) 110:159–73. doi: 10.1161/CIRCRESAHA.111.243162 PMC3690135 22223212

[91] Adamo L Rocha-Resende C Prabhu SD Mann DL. Reappraising the role of inflammation in heart failure. Nat Rev Cardio. (2020) 17:269–85. doi: 10.1038/s41569-019-0315-x 31969688

[92] Shimizu Y Polavarapu R Eskla KL Pantner Y Nicholson CK Ishii M. Impact of lymphangiogenesis on cardiac remodeling after ischemia and reperfusion injury. J Am Heart Assoc. (2018) 7:e009565. doi: 10.1161/JAHA.118.009565 30371303 PMC6404883

[93] Liu X de la Cruz E Gu X Balint L Oxendine-Burns M Terrones T. Lymphoangiocrine signals promote cardiac growth and repair. Nature. (2020) 588:705–11. doi: 10.1038/s41586-020-2998-x PMC7770123 33299187

[94] Hartikainen J Hassinen I Hedman A Kivela A Saraste A Knuuti J. Adenoviral intramyocardial VEGF-DDeltaNDeltaC gene transfer increases myocardial perfusion reserve in refractory angina patients: a phase I/IIa study with 1-year follow-up. Eur Heart. (2017) 38:2547–55. doi: 10.1093/eurheartj/ehx352 PMC5837555 28903476

[95] Wang QL Wang HJ Li ZH Wang YL Wu XP Tan YZ. Mesenchymal stem cell-loaded cardiac patch promotes epicardial activation and repair of the infarcted myocardium. J Cell Mol Me. (2017) 21:1751–66. doi: 10.1111/jcmm.2017.21.issue-9 PMC5571540 28244640

[96] Trincot CE Xu W Zhang H Kulikauskas MR Caranasos TG Jensen BC. Adrenomedullin induces cardiac lymphangiogenesis after myocardial infarction and regulates cardiac edema via connexin 43. Circ Re. (2019) 124:101–13. doi: 10.1161/CIRCRESAHA.118.313835 PMC6318063 30582443

